# Kangquan Recipe Regulates the Expression of BAMBI Protein via the TGF-*β*/Smad Signaling Pathway to Inhibit Benign Prostatic Hyperplasia in Rats

**DOI:** 10.1155/2019/6281819

**Published:** 2019-05-02

**Authors:** Wenfan Chen, Xiaoqing Huang, Axiang Peng, Tingting Chen, Renzhi Yang, Yuanpeng Huang, Zongbao Yang, Shengyan Xi

**Affiliations:** ^1^Department of Traditional Chinese Medicine, Zhongshan Hospital, Xiamen University, Xiamen 361004, Fujian Province, China; ^2^Department of Traditional Chinese Medicine, School of Medicine, Xiamen University, Xiamen 361102, Fujian Province, China

## Abstract

**Background:**

Kangquan Recipe (KQR) is a traditional Chinese medicine compound made by our research group for the treatment of benign prostatic hyperplasia (BPH). Whether KQR can treat BPH as a single drug or play a role in the treatment of BPH in combination therapy needs further study.

**Aim of the Study:**

To investigate the effect of KQR on the expression of TGF-*β*/Smad signaling pathway-related factors in rats with BPH. In-depth analysis revealed the relevant signal transduction mechanism by which KQR acts to treat BPH.

**Materials and Methods:**

Forty-eight male Sprague-Dawley rats were randomly divided into six groups of 8 rats each. In addition to the control group, 40 rats were castrated and then injected with testosterone propionate to form a prostatic hyperplasia model. After 30 days, three groups received different concentrations of KQR (14 g/kg, 7 g/kg, and 3.5 g/kg), and the finasteride group received 0.5 mg/kg finasteride. The BPH group and the control group received the same volume of saline. All groups were treated for a total of 30 days. Rat body weight, prostate volume, wet weight, index, histology, and the mRNA and protein levels of TGF-*β*, TGF-*β*R1, TGF-*β*R2, p-Smad2, p-Smad3, BAMBI, E-cadherin, and N-cadherin in the prostate tissue were measured after the end of treatment.

**Results:**

Compared with the control group, the BPH group had increased prostate wet weight, volume, and index, and the histology showed significant BPH. Compared with the BPH group, the three KQR groups and the finasteride group all had varying levels of reduction in the prostate wet weight, volume, and index of the prostate and varying degrees of improvement in the histological manifestations of BPH. KQR downregulates the mRNA and/or protein expression of TGF-*β*, TGF-*β*R1, TGF-*β*R2, p-Smad2, p-Smad3, and N-cadherin protein in prostate tissue and increases the mRNA and protein expression of BAMBI and E-cadherin protein.

**Conclusions:**

In the model of BPH induced by testosterone propionate after castration, KQR can inhibit the conduction of the TGF-*β*/Smad signaling pathway by upregulating the expression of BAMBI protein and reversing EMT in rat prostate tissue.

## 1. Introduction

Benign prostatic hyperplasia (BPH) is a common disease in elderly men, with lower urinary tract symptoms (LUTS) caused by hyperplasia of the prostatic epithelium and stromal cells [1]. At present, the main therapeutic drugs for BPH are 5*α*-reductase inhibitors and *α*-blockers [2, 3], such as finasteride and terazosin. However, they can cause adverse reactions such as fatigue, hypotension, ejaculation disorders, sexual dysfunction, and an increased risk of prostate fibrosis [3, 4]. Surgical treatment of BPH requires strict surgical indications and carries with it the inevitable complications and risks of surgery and the possibility of recurrence. In fact, the number of patients undergoing surgery for BPH is gradually decreasing [5]. Therefore, it is particularly important to find a therapeutic drug that can effectively treat BPH with few adverse reactions.

Traditional Chinese medicine (TCM) treatment of diseases, including BPH, has been reported [6, 7]. In this study, Kangquan Recipe (KQR) has a unique effect in treating BPH, with mild effects and small side effects. Clinical studies have shown that [8] KQR can effectively reduce International Prostate Symptom Score (I-PSS) scores, residual urine volume (RUV), and total prostatic volume(TPV) and improve the maximum urinary flow rate (Qmax) and average urine flow rate (Qave) levels in patients with BPH. Previous experimental studies found that [9] the mechanism of KQR treatment of BPH may be to regulate the balance of plasma testosterone and estradiol and reduce the expression of proliferating cell nuclear antigen (PCNA) mRNA to inhibit prostate cell proliferation. However, the potential mechanism by which KQR acts in the treatment of BPH remains to be further studied. Recently, the influence of the transforming growth factor-*β* (TGF-*β*)/suppressor of mothers against decapentaplegic (Smad) signaling pathway on BPH has attracted the attention of many scholars.

In BPH, activation of the TGF-*β*/Smad signaling pathway occurs when TGF-*β*1 binds with the receptors on the envelope and then combines with Smad to form a complex [10]. The Smad complex enters the nucleus and binds to the Smad binding element in the promoter region of epithelial-mesenchymal transition (EMT)-related genes, regulating the expression of *α*-smooth muscle actin connective tissue growth factor and other EMT-related target genes, thereby causing EMT in prostate tissue [11]. Therefore, the TGF-*β*/Smad signaling pathway is considered one of the key pathways leading to EMT in prostate epithelial cells.

In the process of the TGF-*β* family members binding to the receptors, there is a pseudoreceptor, namely, bone morphogenetic protein and activin membrane-bound inhibitor (BAMBI). It does not possess kinase activity due to the lack of a serine/threonine kinase domain in the intracellular region [12]. However, it has a similar structure to the extracellular domain of TGF-*β* type I receptor, meaning that it can compete with type I receptors and bind to type II receptors to form the ligand-receptor complex, thereby blocking the transduction of the signal along the TGF-*β*/Smad signaling pathway [13, 14].

Therefore, this study aimed to observe the regulation by KQR of the expression of TGF-*β*/Smad signaling pathway-related factors in prostate tissue and to further explore the mechanism of action of KQR, which is helpful in elucidating the related signal transduction mechanism of BPH to provide a basis for clinical treatment. At the same time, we searched the existing literature and found that few studies have investigated the effect of BPH on the TGF-*β*/Smad signaling pathway. This study is a prospective study of traditional Chinese medicine. The aims of this study were to explore multitarget and multichannel regulation by a traditional Chinese medicine, to promote the in-depth application of traditional Chinese medicine to treat BPH and to form an effective systematic and standardized treatment plan.

## 2. Materials and Methods

### 2.1. Experimental Animals

In total, 48 male Sprague-Dawley (SD) rats (200 ± 20 g) were purchased from Shanghai Slack Laboratory Animals Co., Ltd. These animals were placed in cages in the animal room of Xiamen University Medical College [License No. SYXK (Min) 2013-0006]. The rats were kept under the following conditions: room temperature 20-22°C, relative humidity 65-70%, 12 h natural light dark cycle, and ad libitum food [Beijing Huakang Biotechnology Co., Ltd., Beijing Feed Certificate (2014) 06057] and water. The study was reviewed and approved by the Animal Ethics Committee of Xiamen University and conducted according to the guidelines for the care and use of laboratory animals.

### 2.2. Experimental Drug Preparation Techniques

The medicinal components of KQR [Morinda officinalis (voucher number: 170713), Pheretima aspergillum (voucher number: 170814), Eupolyphaga sinensis Walker (voucher number: 1703310162), Rheum palmatum (voucher number: 170526), Trachelospermum jasminoides (voucher number: 170601), and Cinnamomum cassia Presl (voucher number: 171117)] were purchased from Yanlaifu Co., Ltd. (production license number: Fujian 20112046) and approved by Professor Yingkun Qiu (Xiamen University, China). All the herbals are stored in the Experimental Center of Traditional Chinese Medicine of Medical College of Xiamen University. Then, 15 g of Morinda officinalis, 15 g of Pheretima aspergillum, 10 g of Eupolyphaga sinensis Walker, 5 g of Rheum palmatum, 20 g of Trachelospermum jasminoides, 5 g of Cinnamomum cassia Presl., and 700 mL of distilled water were directly mixed. The mixture was left to soak in the decocting container for 30 minutes and then boiled with high heat, simmered for 30 minutes, and filtered through 8 layers of filter gauze. Next, 450 mL of distilled water was added to the decocting container and cooked for another 30 minutes at low heat. The two combined filtrates were concentrated to 50 mL at 58°C in a rotary evaporator (RE-52AA, Shanghai Yarong), which corresponds to a high dose of 1.4 g/mL of the crude drug. A middle dose of 0.7 g/mL and a low dose of 0.35 g/ml were prepared by dilution with distilled water.

### 2.3. HPLC

Chemical analysis of KQR was performed using a liquid chromatograph model Shimadzu LC-20A (Shimadzu Corporation, Japan). The separation column model is YMC-Pack ODS column (YMC, Japan), and the mobile phase is 0.1% phosphoric acid aqueous solution (A)-acetonitrile (B), 5-45% acetonitrile gradient system, elution time 0-60 minutes, and 100% B, 60-70 minutes. The flow rate was 1 mL/min, and the test solution injection volume was 20 *μ*L.

### 2.4. Main Reagents

The reagents used were as follows. Finasteride tablets (product code: NO22063) was supplied by Merck Sharp & Dohme Limited, UK. Testosterone propionate injection (batch number: 170512) was provided by China Ningbo Second Hormone Factory. TRIZOL reagent (3070-250) was provided by Beijing Tian Enze Gene Technology Co., Ltd., China. ReverTra Ace qPCR RT Master Mix (FSQ-201) was supplied by the TOYOBO Corporation of Japan. FS Universal SYBR Green Master (4913914001) was supplied by ROCHE, Switzerland. Anti-TGF *β*R1 antibody (product code: ab31013), anti-TGF-*β*R2 antibody (product code: ab186838), anti-BAMBI antibody (product code: ab203070), anti-E-cadherin antibody (product code: ab133597), and N-cadherin (product code: ab18203) were provided by Abcam, UK. Anti-TGF *β*1 (product code: ARG55096), p-Smad2 (product code: ARG51796), and p-Smad3 (product code: ARG51797) were purchased from Arigo Company, China.

### 2.5. Instruments

The instruments used are listed below. The precision balance (PL601-L) was produced by METTLER TOLEDO, Switzerland. The Intelligent Biomicroscope (BX53) was produced by the Olympus Corporation of Japan. The paraffin slicer (RM2235), embedding machine (EG1150H), and fully automatic tissue dewatering machine (ASP200S) were produced by Leica, Germany. The gel electrophoresis apparatus (PowerPac Basic) and the electrophoresis tank (Mini-PROTEAN) were manufactured by the Bio-Rad Corporation in the United States. The fully automatic chemiluminescence image analysis system (5200S type) was produced by Tanon Company, China. The PCR instrument (Model ABI7500) was produced by Applied Biosystems, USA. The PRO Multi-Function Label (M200) was produced by Tecan, Switzerland.

### 2.6. Establishment of Benign Prostatic Hyperplasia Animal Model

The rat model of BPH was established according to the protocol used in previous experiments [9]. After one week of adaptation to the environment, rats in all groups except the control group were anesthetized via abdominal injection of chloral hydrate (3 mL/kg), and the scrotums of the rats were cut layer by layer under sterile conditions. The testis and epididymis were exfoliated to avoid the effects of residual testosterone in the body. The stump was ligated and then sutured layer by layer. The cages were kept clean and dry to prevent infection of the incision. After a week of recovery, the animals were injected subcutaneously with testosterone propionate at a dose of 3.5 mg/kg once daily for 30 days. The control group was injected subcutaneously with physiological saline.

### 2.7. Experimental Design

A total of forty-eight SD male rats were randomly divided into 6 groups by random number table method. 8 rats were as the control group, and the other 40 prostate hyperplasia rat models were randomly divided into the following groups, with 8 rats in each group: KQR high dose group, KQR middle dose group, KQR low dose group, finasteride group (positive control), and BPH group. These groups of rats were injected subcutaneously with 3.5 mg/kg testosterone propionate daily, and the control group was injected with the same amount of normal saline for 30 consecutive days. According to the equivalent dose conversion formula for humans and rats, the KQR high dose group received 14 g/kg (corresponding to 12 times the clinical dose); the KQR middle dose group received 7 g/kg (equivalent to 6 times the clinical dose); the KQR low dose group received 3.5 g/kg (equivalent to 3 times the clinical dose); and the finasteride control group received 0.5 mg/kg (equivalent to 6 times the clinical dose); the medicine was dissolved in 10 mL/kg of normal saline immediately before use. In the control group and the BPH group, only 10 mL/kg of normal saline was administered. The administration volume was 1 mL/100 g, and it was administered intragastrically once a day for 30 consecutive days (during the administration period, the body weight was measured once a week, and the amount was adjusted accordingly).

### 2.8. Measurement of Rat Body Weight, Prostate Volume, Wet Weight, and Index

After 4 weeks of treatment, the body weight of the rats was measured with a balance. After the rats were euthanized, the wet weights of the bilateral ventral lobes of the prostate tissue were measured, and the prostate volume was measured by the volumetric method. The prostate index (PI = prostate wet weight/body weight × 100%) was calculated.

### 2.9. Prostate Tissue Pathological Changes

A portion of rat prostate tissue was immersed in 10% formalin for fixation, dehydrated, and embedded in paraffin. Hematoxylin and eosin (H&E) staining was performed after the tissue was cut into 4-micron-thick slices, and pathological changes in the rat prostate tissue were observed under a microscope.

### 2.10. Western Blotting Detected the Protein Expression Levels of TGF-*β*, TGF-*β*R1, TGF-*β*R2, pSmad2, p-Smad3, BAMBI, E-Cadherin, and N-Cadherin in Rat Prostate Tissue

Western blotting was performed with reference to the method in Xi et al. [15], in which the following antibodies were used: rabbit anti-anti-TGF *β*1 antibody (1:1000 dilution); rabbit anti-anti-TGF-*β* receptor 1 antibody (1:1000 dilution); rabbit anti-anti-TGF-*β* receptor 2 antibody (1:1000 dilution); rabbit anti-anti-Smad 2 antibody (1:1000 dilution); rabbit anti-anti-Smad 3 antibody (1:1000 dilution); rabbit anti-anti-BAMBI antibody (1:1000 dilution); rabbit anti-E-cadherin antibody (1:1000 dilution); rabbit anti-N-cadherin antibody (1:1000 dilution); and murine anti-GAPDH monoclonal antibody (1:5000 dilution).

### 2.11. qRT-PCR Was Used to Detect the mRNA Expression of TGF-*β*, TGF-*β*R1, BAMBI, Smad2, E-Cadherin, and N-Cadherin in Rat Prostate Tissue

The qRT-PCR was performed with reference to the protocol in Xi et al. and previous experiments [9, 15]. The primers were synthesized by Shanghai Shenggong Co., Ltd., and the primer sequences were as follows: GAPDH: F-ACCA CAGT CCAT GCCA TCAC, R-TCCA CCAC CCTG TTGC TG, 168 bp; TGF-*β*: F-CATT GCTG TCCC GTGC AGA, R-AGGT AACG CCAG GAAT TGTT GCTA, 103 bp; TGF*β*R1: F-CACA CAGT CAGT GCGG TGAG AG, R-AGAG GCCC ACAA GAGT TTCA ACA, 88 bp; BAMBI: F-GCAA TTAC CGAG GACT GCAT GA, R-TCCT GCAC CTTA GTGA TGAG GTTT C, 112 bp; Smad2: F-TTAC AGAT CCAT CGAA CTCG GAGA, R-CACT TAGG CACT CGGC AAAC AC, 150 bp; E-cadherin: F-CACA CTGA TGGT GAGG GTAC AAGG, R-GGGC TTCA GGAA CACA TACA TGG, 123 bp; N-cadherin: F-GACT GCAC CGAC GTAG ACAG GA, R-ATCC ATAC CACG AACA TGAG GACA, 116 bp. Each experiment was repeated three times.

### 2.12. Statistical Analysis

The parameter data were expressed as means ± SD ($\bar{x}\;\;$± s). Experimental data were analyzed by using GraphPad Prism 7 software (GraphPad Software Inc., La Jolla, United States). Statistical significance was determined by using one-way analysis of variance [ANOVA]. Differences of* P*<0.05 were considered significant.

## 3. Results

### 3.1. Analysis of KQR

**Figure 1:**
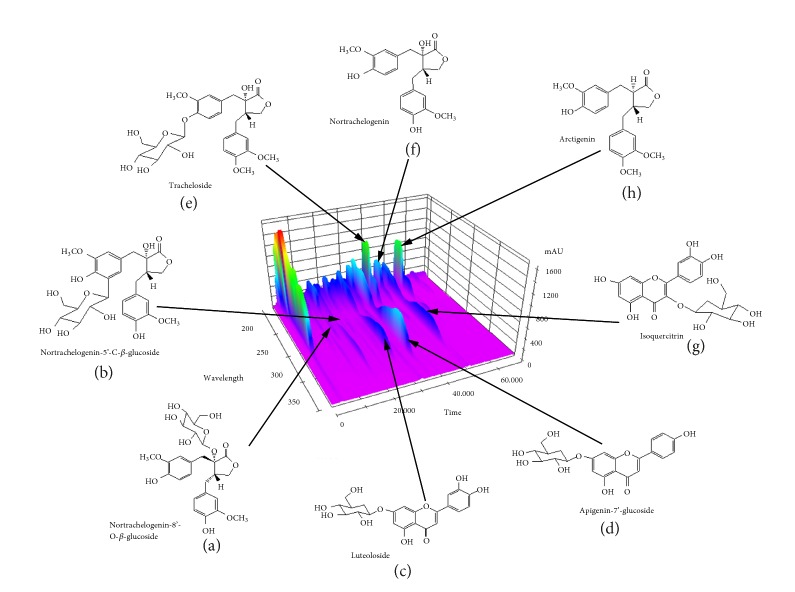
Chemical structure of KQR active ingredients identified by HPLC. Nortrachelogenin-8′-O-*β*-glucoside (a), Nortrachelogenin-5′-C-*β*-glucoside (b), Luteoloside (c), Apigenin-7′-glucoside (d), Tracheloside (e), Nortrachelogenin (f), Isoquercitrin (g), and Arctigenin (h).

As shown in Figure 1, KQR was separated with High Performance Liquid Chromatography (HPLC) system and its chromatographic fingerprinting established. Comparing the retention time and UV spectra with reference samples, the following 8 major chemical compositions were identified: Nortrachelogenin-8′-O-*β*-glucoside (peak 1, Rt = 15.01 min), Nortrachelogenin-5′-C-*β*-glucoside (peak 2, Rt = 25.78 min), Luteoloside (peak 3, Rt = 28.69 min), Apigenin-7′-glucoside (peak 4, Rt = 36.23 min), Tracheloside (peak 5, Rt = 40.81 min), Nortrachelogenin (peak 6, Rt = 46.27 min), Isoquercitrin (peak 7, Rt = 49.05 min), and Arctigenin (peak 8, Rt = 54.91 min).

### 3.2. Effect of KQR on Wet Weight, Prostate Volume, and PI in Rats

**Figure 2:**
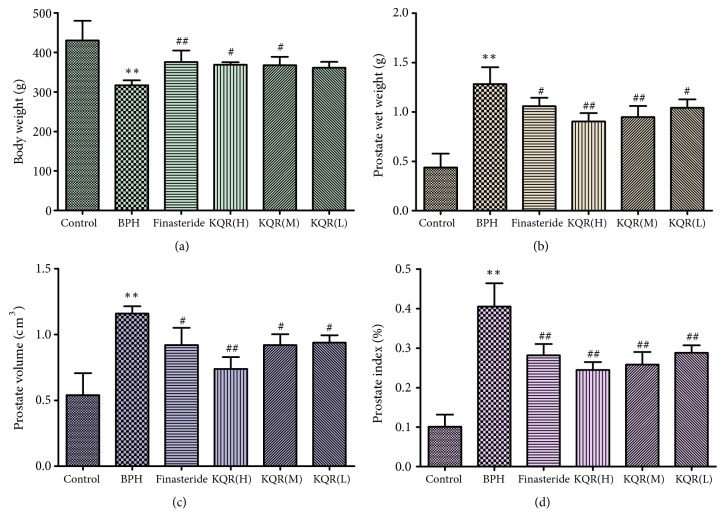
Effects of KQR on body weight, prostate wet weight, volume, and PI in rats. Data were represented as mean ± SD (n = 6 mice per group). Statistical analysis: *∗P*<0.05; *∗∗P*<0.01 compared with the normal group; ^#^*P*<0.05; ^##^*P*<0.01 compared with the model group.

As shown in Figures 2(a)–2(d), compared with the control group, the weight, prostate wet weight, prostate volume, and PI were significantly different in the BPH group (*P*<0.01). Compared with the BPH group (Figures 2(b)–2(d)), the three KQR group and finasteride (control) group had significantly lower prostate wet weights, prostate volumes, and PI values (*P*<0.05 or* P*<0.01). Compared with the finasteride group, the KQR high and middle dose groups had no significant differences in prostate wet weight, prostate volume, and PI values (*P*>0.05). KQR could significantly reduce the prostate wet weight, prostate volume and PI of rats with BPH; its effect had a positive relationship with the drug concentration.

### 3.3. Effect of KQR on the Pathological Morphology of Prostate Tissue in Rats with BPH

**Figure 3:**
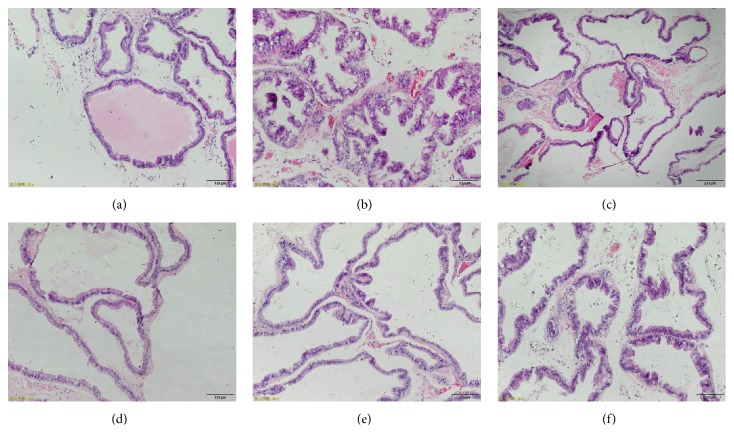
Effects of KQR on pathology of prostate tissue in rats. Sections were stained with hematoxylin and eosin (H&E) and viewed at a magnification of ×200. (a) Control group; (b) BPH group; (c) finasteride group (0.5 mg/kg); (d) KQR high dose group (14 g/kg); (f) KQR middle dose group (7 g/kg); (f) KQR low dose group (3.5 g/kg).

Pathological observation was conducted on prostate tissue in each group. The prostate gland of the control group (Figure 3(a)) showed no hyperplasia, and the glandular epithelial cells were mostly cubic and arranged in a single layer. And interstitial and fibrous tissue content did not increase. The prostate gland of the BPH group (Figure 3(b)) had proliferation, with an increased number of glandular epithelial cells and an increased volume with high columnarity. The cells were arranged in a single layer or multiple layers, and the glandular epithelial cell layer formed folds into the cavity, showing papillary processes; interstitial fibrous tissue and smooth muscle tissue increase. In the finasteride group (Figure 3(c)), the prostate gland was dilated, some epithelial cells still proliferated, but the papillary processes were significantly reduced and interstitial fibrous tissue was not obvious. Prostate hyperplasia was significantly alleviated in the KQR high dose group (Figure 3(d)), and epithelial cells were mostly in a single layer with few papillary processes. And interstitial fibrous tissue and smooth muscle tissue were significantly reduced. In the KQR middle dose group (Figure 3(e)), the prostate gland was shrunken, the glandular epithelial cells were slightly proliferated, the wrinkles were reduced, and only a small part showed papillary processes, a small amount of interstitial tissue, and vascular fibrous tissue. The prostate glands of the KQR low dose group (Figure 3(f)) were slightly dilated, and the folds of the glandular epithelial cells into the cavity were slightly reduced; the epithelial cells were enlarged and closely arranged. And partial interstitial fibrous tissue and smooth muscle tissue were showed.

### 3.4. Effects of KQR on the Protein Expression of TGF-*β*, TGF-*β*R1, TGF-*β*R2, p-Smad2, p-Smad3, BAMBI, E-Cadherin, and N-Cadherin in the Prostate Tissue of Rats with BPH

**Figure 4:**
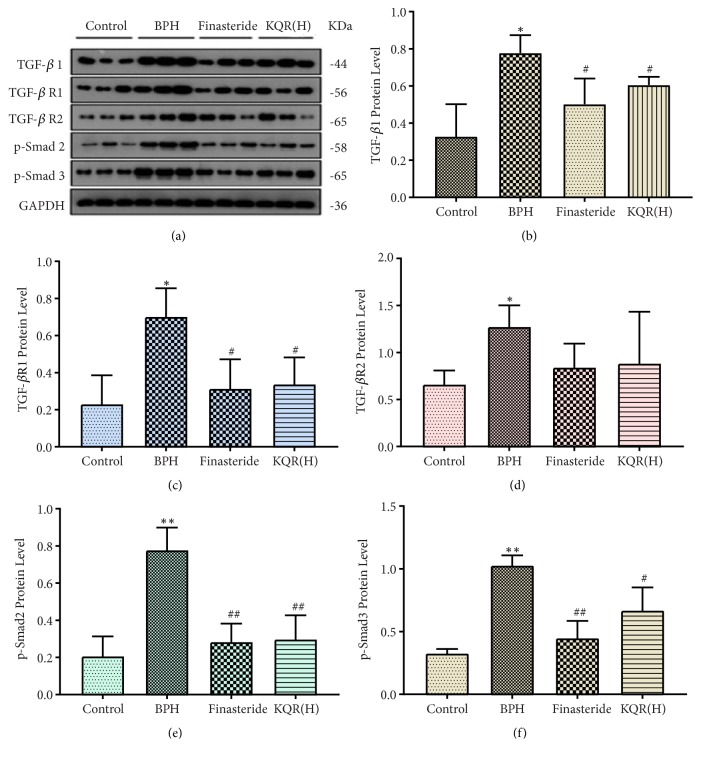
Effects of KQR on the protein expression of TGF-*β*, TGF-*β*R1, TGF-*β*R2, p-Smad2, and p-Smad3 in the prostate tissue. Data were represented as mean ± SD (n = 6 mice per group). Statistical analysis: *∗P*<0.05; *∗∗P*<0.01 compared with the control group; ^#^*P*<0.05; ^##^*P*<0.01 compared with the BPH group.

As shown in Figure 4, the expression levels of TGF-*β*, TGF-*β*R1, TGF-*β*R2, p-Smad2, and p-Smad3, which were related to the TGF-*β*/Smad signaling pathway, were upregulated in the BPH group compared with the control group (*P*<0.05 or* P*<0.01). The expression levels of TGF-*β*, TGF-*β*R1, TGF-*β*R2, p-Smad2, and p-Smad3 were significantly decreased after finasteride treatment (*P*<0.05 or* P*<0.01). Among the KQR groups, the high dose group also downregulated the expression levels of TGF-*β*, TGF-*β*R1, TGF-*β*R2, p-Smad2, and p-Smad3 relative to the model group (*P*<0.05 or* P*<0.01) (see the Supplementary Figure 1), and there was no significant difference in protein levels compared with the finasteride groups (*P*>0.05). However, as the concentration of KQR decreased, its downregulation effects were weakened.

**Figure 5:**
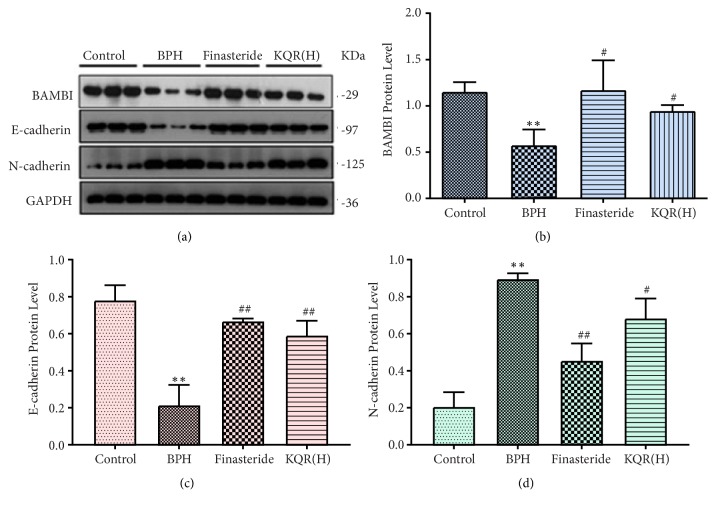
Effects of KQR on the protein expression of BAMBI, E-cadherin, and N-cadherin in the prostate tissue. Data were represented as mean ± SD (n = 6 mice per group). Statistical analysis: *∗P*<0.05; *∗∗P*<0.01 compared with the control group; ^#^*P*<0.05; ^##^*P*<0.01 compared with the BPH group.

BAMBI is a pseudoreceptor in the TGF-*β* signaling pathway, which is structurally similar to TGF-*β*R1 and competitively binds to TGF-*β*R2, thereby blocking the TGF-*β*/Smad signaling pathway. As shown in Figure 5, compared with the control group, the BPH group had decreased expression of BAMBI (*P*<0.05). Interestingly, the expression of BAMBI was significantly upregulated after KQR high doses or finasteride treatment (*P*<0.05). As the concentration of KQR decreased, the level of upregulation decreased (see the Supplementary Figure 2). E-cadherin and N-cadherin are epithelial-mesenchymal transition (EMT)-related factors. Compared with the control group, the BPH group had increased expression of N-cadherin and decreased expression of E-cadherin (*P*<0.05). KQR high doses or finasteride were able to downregulate the expression of N-cadherin and upregulate the expression of E-cadherin (*P*<0.05), but as the concentration of KQR increased, its effect is strengthened (see the Supplementary Figure 2).

### 3.5. Effects of KQR on the mRNA Expression of TGF-*β*, TGF-*β*R1, BAMBI, Smad2, E-Cadherin, and N-Cadherin in the Prostate Tissue of Rats with BPH

**Figure 6:**
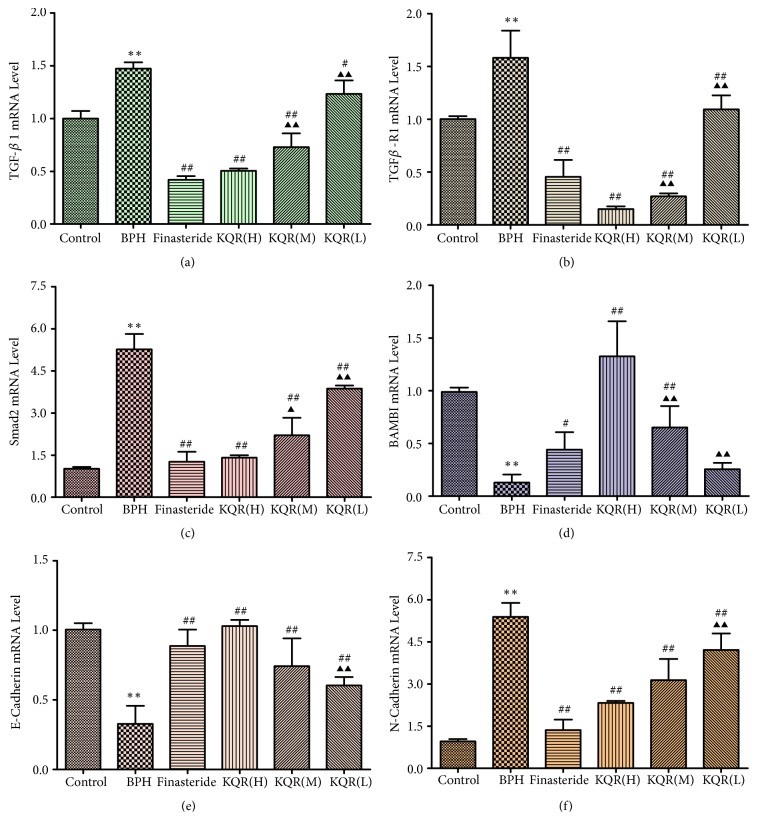
Effects of KQR on the mRNA expression of TGF-*β*, TGF-*β*R1, BAMBI, Smad2, E-cadherin, and N-cadherin in the prostate tissue. Data were represented as mean ± SD (n = 6 or 7 mice per group). Statistical analysis: *∗P*<0.05; *∗∗P*<0.01 compared with the control group; ^#^*P*<0.05, ^##^*P*<0.01 compared with the BPH group; ^▲^*P*<0.05; ^▲▲^*P*<0.01 compared with the KQR high dose group.

Figure 6 shows that compared with the control group, the BPH group had increased mRNA expression levels of TGF-*β*, TGF-*β*R1, p-SMAD2, and N-cadherin (Figures 6(a)–6(c), and 6(f)) and the expression levels of BAMBI and a decreased mRNA expression level of E-cadherin (*P*<0.01) (Figures 6(d) and 6(e)). Compared with the BPH group, the KQR and finasteride groups had decreased mRNA expression levels of TGF-*β*, TGF-*β*R1, Smad2, and N-cadherin and increased expression mRNA expression levels of BAMBI and E-cadherin (*P*<0.05 or* P*<0.01). Compared with the finasteride group, the KQR middle and low dose groups had no significant differences in the level of BAMBI mRNA expression, and the KQR high and middle dose groups had no significant different in mRNA expression level of E-cadherin (*P*>0.05). Compared with the KQR high dose group, the KQR middle and low dose groups had significantly difference mRNA expression levels of BAMBI, Smad2, N-cadherin, and E-cadherin (*P*<0.05 or* P*<0.01). These results suggest that KQR could decrease the mRNA expression of TGF-*β*, TGF-*β*R1, Smad2, and N-cadherin and increase the mRNA expression of BAMBI and E-cadherin in the prostates of rats with BPH. The relationship was dose-dependent.

## 4. Discussion

BPH is a common disease in older men. With the increase in the aging population, the BPH has attracted increasing attention [16]. There is a wealth of experience with using traditional Chinese medicine in the treatment of this disease. Traditional Chinese medicine states that the pathological features of BPH are based on kidney deficiency. The incidence is caused by hot and humid bets, qi stagnation, blood stasis, and heat accumulation [17]. KQR consists of morinda officinalis, pheretima aspergillum, eupolyphaga sinensis Walker, rheum palmatum, trachelospermum jasminoides, and cinnamomum cassia Presl.

Morinda officinalis has anti-inflammatory and antioxidation activities [18], inhibiting the production of prostaglandin E2 and tumor necrosis factor-*α* (TNF-*α*) is beneficial for the treatment of BPH [19]. Pheretima aspergillum has a variety of beneficial pharmacological activities, including diuretic, fibrinolytic, anticoagulant, anti-inflammatory, and antioxidation [20]. Eupolyphaga sinensis Walker have anticoagulant, antithrombotic, and antitumor effects [21]. Rheum palmatum has been shown to have anti-inflammatory, antioxidation, anticancer, and improved renal function [22]. The pharmacological properties of the trachelospermum jasminoides include anti-inflammatory, analgesic, antitumor, antiviral, and antibacterial activities [23]. The combination of various medicines, both attacking and supplementing, tonifies the kidneys and replenishing the qi, promoting blood circulation, and clearing and dissipating the heat. The results of this experiment show that KQR can reduce the PI value, prostate volume, and wet weight in rats. Tissue sections also confirmed that KQR can significantly inhibit prostate gland dilation in rats. This indicates that KQR has a good therapeutic effect on rats with BPH. This finding further proves the effectiveness of KQR in the treatment of BPH.

It is worth mentioning that eight active substances in the KQR component that are beneficial for the treatment of BPH have been detected. Specifically, Nortrachelogenin, Nortrachelogenin-5′-C-*β*-glucoside, and Nortrachelogenin-8′-O-*β*-glucoside are a group of plant-derived polyphenolic compounds that induce cell death and inhibit cancer cell proliferation and reduce hormone dependence. The risk of sexual cancer [24]; Luteoloside has anti-inflammatory, antioxidant, and antitumor effects [25]; Apigenin-7′-glucoside has antioxidant, anti-inflammatory, and antitumor activities [26]; not only has Trachelogenin liver protection, but also antitumor viral activity is involved in inhibiting the proliferation of tumor cells [27]; both in vivo and in vitro studies of Isoquercitrin have been shown to treat cancer, cardiovascular disease, and allergic reactions [28]; Arctigenin studies have shown strong antiprostate cancer activity [29].

BPH is a disease that most men face. The incidence of BPH in men aged 61-70 is 70%, and the incidence after 80 years is 90% [30]. TGF-*β* plays an important role in the development of BPH. Excessive expression of TGF-*β* can induce local vascular formation and inflammatory responses and can also increase the incidence of BPH [31, 32]. Studies have shown that the expression of TGF-*β*1 is elevated in BPH [33], and the high expression level of TGF-*β* is also associated with poor clinical outcomes [34]. In prostatic hyperplasia cell lines, the expression level of TGF-*β* is associated with the migration of BPH-1 cells [35]. TGF-*β* is able to exert its biological effects by binding to receptors type 1 and 2 [36]. TGF-*β*R1 and TGF-*β*R2 are important factors in TGF-*β*/Smad signaling [37]. The TGF-*β* receptors contain a serine/threonine kinase that is directly involved in TGF-*β* signaling [38]. The experimental results were as expected: KQR can downregulate the expression level of TGF-*β*1, and the expression levels of TGF*β*R-1 and TGF*β*R-2 are also decreased. One of the mechanisms by which KQR treats BPH may be through the inhibition of the TGF-*β*/Smad signaling pathway.

The main purpose of this study was to analyze the effect of KQR on TGF-*β*/Smad signaling in rat prostate tissue. In the Smad family, Smad2 and Smad3 are receptor-activated Smads (R-SMADs) that can be phosphorylated and activated by TGF-*β* binding to type 1 and type 2 receptors [39]. Phosphorylated Smad is responsible for transducing TGF-*β* signaling from the cell membrane to the nucleus, where the transcription of the target gene is either induced or inhibited, thereby completing the signal transduction [40]. Phosphorylation of Smad2 or Smad3 (p-Smad) represents the activation of the TGF-*β*/Smad signaling pathway. The abnormal expression of the Smad protein has been confirmed to be closely related to the occurrence and development of BPH [41–43]. Our data showed that the levels of p-Smad2 and p-Smad3 and the levels of Smad2 mRNA in the prostate tissue of rats treated with KQR were lower than those in the model group. This may be one of the reasons for the decrease in the activation of the TGF-*β*/Smad signaling pathway. Furthermore, KQR reduced the expression of TGF-*β*1 and inhibited the phosphorylation of Smad2 and Smad3, blocking the TGF-*β*/Smad signaling pathway and thereby inhibiting the development of BPH. Next, we investigated the mechanism by which KQR blocked the activation of the TGF-*β*/Smad signaling pathway.

The BAMBI protein is a transmembrane glycoprotein and a pseudoreceptor of the TGF-*β*/Smad signaling pathway [44]. Studies have shown that inhibition of BAMBI expression in colorectal cancer can upregulate TGF-*β* signaling, leading to elevated levels of p-Smad2 and p-Smad3 [45]. Yan et al. found that the complex formed by BAMBI protein, Smad7, and TGF-*β*1 receptor can prevent the phosphorylation of Smad3 and block the TGF-*β*/Smad signaling pathway [46]. In addition, there is a therapeutic effect on the development of prostate cancer [47]. Although the BAMBI protein has been studied in many diseases such as tumors and tissue fibrosis [48, 49], the influence of traditional Chinese medicine on BAMBI in BPH is poorly understood. In the present study, we found that BAMBI mRNA and protein were expressed in rat prostate specimens and upregulated with KQR treatment. These data indicate that overexpression of BAMBI protein inhibits TGF-*β* signaling and prevents the activation and phosphorylation of Smad2 and Smad3.

In EMT, the expression levels of molecular markers on epithelial cells such as E-cadherin are decreased, while the expression levels of molecular markers in interstitial cells such as N-cadherin are increased [50]. Alonso-Magdalena et al. also found that cells proliferating in BPH tissue are derived from EMT [51]. In recent years, studies have shown [52] that EMT occurs in BPH cells and is associated with the TGF-*β*/Smad signaling pathway. These studies suggest that EMT may be involved in the development of BPH and play an important role. Our results indicate that, in prostate tissue overexpressing BAMBI protein, the protein expression levels of E-cadherin are upregulated, while the protein expression level of N-cadherin is downregulated. This suggests that KQR can regulate the expression of BAMBI and thereby intervention of the TGF-*β*/Smad signaling pathway. Upregulation of BAMBI expression may be the main mechanism by KQR to inhibit EMT in prostate tissue. In addition, the results showed that KQR high dose can effectively inhibit the pathological changes of rat prostate tissue, inhibit the expression of TGF, reduce the downstream signal transduction of the TGF-*β*/Smad signaling pathway, and reverse EMT in BPH.

Although our results suggest that KQR can be used as a potential drug for future clinical treatment of BPH, due to the complexity of traditional Chinese medicine ingredients, there may be some shortcomings in this experiment. First, the mechanism by which KQR increases the protein expression of BAMBI remains unclear; and further research is needed to confirm the role of KQR. Second, Chinese medicine formula has multipathway and multitarget effects. Therefore, the TGF-*β*/Smad signaling pathway may not reflect the main mechanisms involved in BPH.

## 5. Conclusion

In summary, this study demonstrates that KQR has the effect of treating BPH in rats. It can upregulate the protein expression of BAMBI and inhibit the expression of TGF-*β*, TGF-*β*R1, TGF-*β*R2, Smad2, p-Smad2, and p-Smad3 related factors to block the transmission of TGF-*β*/Smad signaling pathway and reverse EMT phenomenon in rat prostate tissue. Further research into the mechanism of this formulation will help to demonstrate the potential value of traditional Chinese medicine in the treatment of BPH.

## Data Availability

All the data are available in the manuscript.

## Abbreviations

KQR: Kangquan Recipe
BPH: Benign prostatic hyperplasia
LUTS: Lower urinary tract symptoms
I-PSS: International Prostate Symptom Score
RUV: Residual urine volume
TPV: Total prostatic volume
Qmax: Maximum urinary flow rate
Qave: Average urine flow rate
PCNA: Proliferating cell nuclear antigen
TGF-*β*: Transforming growth factor-*β*
Smad: Suppressor of mothers against decapentaplegic
BAMBI: Bone morphogenetic protein and activin membrane-bound inhibitor
EMT: Epithelial-mesenchymal transition
SD: Sprague-Dawley
PI: Prostate index
HE: Hematoxylin and eosin
TCM: Traditional Chinese medicine.

## Supplementary Materials

Supplementary Materials Supplementary Figure 1: effects of different doses of KQR on protein expression of TGF-*β*, TGF-*β*R1, TGF-*β*R2, p-Smad2, and p-Smad3 in the prostate tissue. Supplementary Figure 2: effects of different doses of KQR on protein expression of BAMBI, E-cadherin, and N-cadherin in the prostate tissue. Data were represented as mean ±SD (n = 6 mice per group). Statistical analysis: ^#^*P*<0.05; ^##^*P*<0.01 compared with the BPH group; ^▲^*P*<0.05; ^▲▲^*P*<0.01 compared with the KQR high dose group.
