# Supplementary material for: Kangquan Recipe Regulates the Expression of BAMBI Protein via the TGF-β/Smad Signaling Pathway to Inhibit Benign Prostatic Hyperplasia in Rats
Source: Evid Based Complement Alternat Med. 2019 May 2;2019:6281819. doi: 10.1155/2019/6281819 (PMC6521302; doi:10.1155/2019/6281819)
Supplement: Supplementary Materials — Supplementary Figure 1: effects of different doses of KQR on protein expression of TGF-β, TGF-βR1, TGF-βR2, p-Smad2, and p-Smad3 in the prostate tissue. Supplementary Figure 2: effects of different doses of KQR on protein expression of BAMBI, E-cadherin, and N-cadherin in the prostate tissue. Data were represented as mean ±SD (n = 6 mice per group). Statistical analysis: #P<0.05; ##P<0.01 compared with the BPH group; ▲P<0.05; ▲▲P<0.01 compared with the KQR high dose group. [file 6281819.f1.docx]

**Supplementary Data**


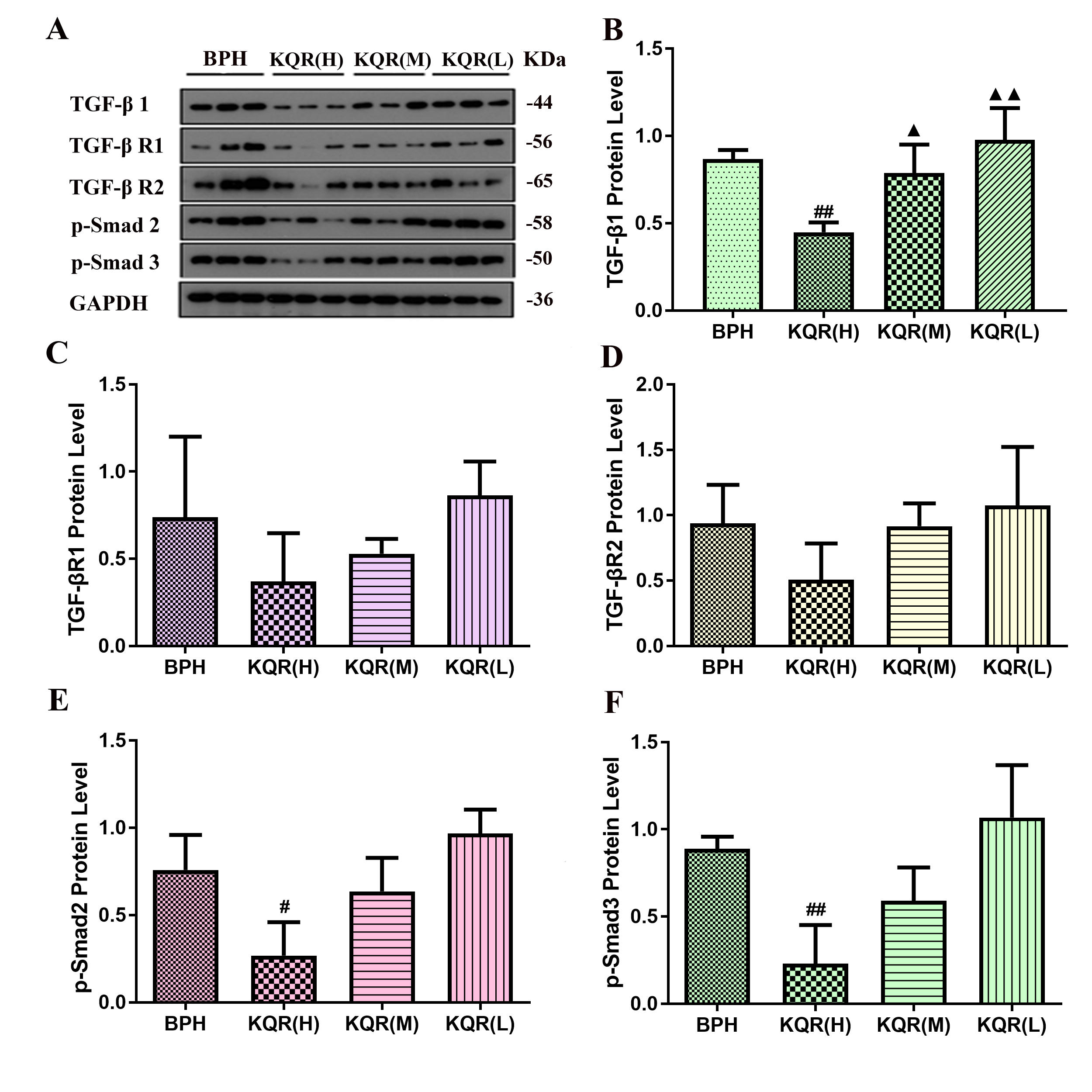


**Fig. S1.** Effects of KQR on the protein expression of TGF-β, TGF-βR1, TGF-βR2, p-Smad2 and p-Smad3 in the prostate tissue. Data were represented as mean ±SD (n=6 mice per group). Statistical analysis: ^#^*P*<0.05, ^##^*P*<0.01 compared with the BPH group; ^▲^*P*<0.05, ^▲▲^*P*<0.01 compared with the KQR high dose group.

**Supplementary Data**


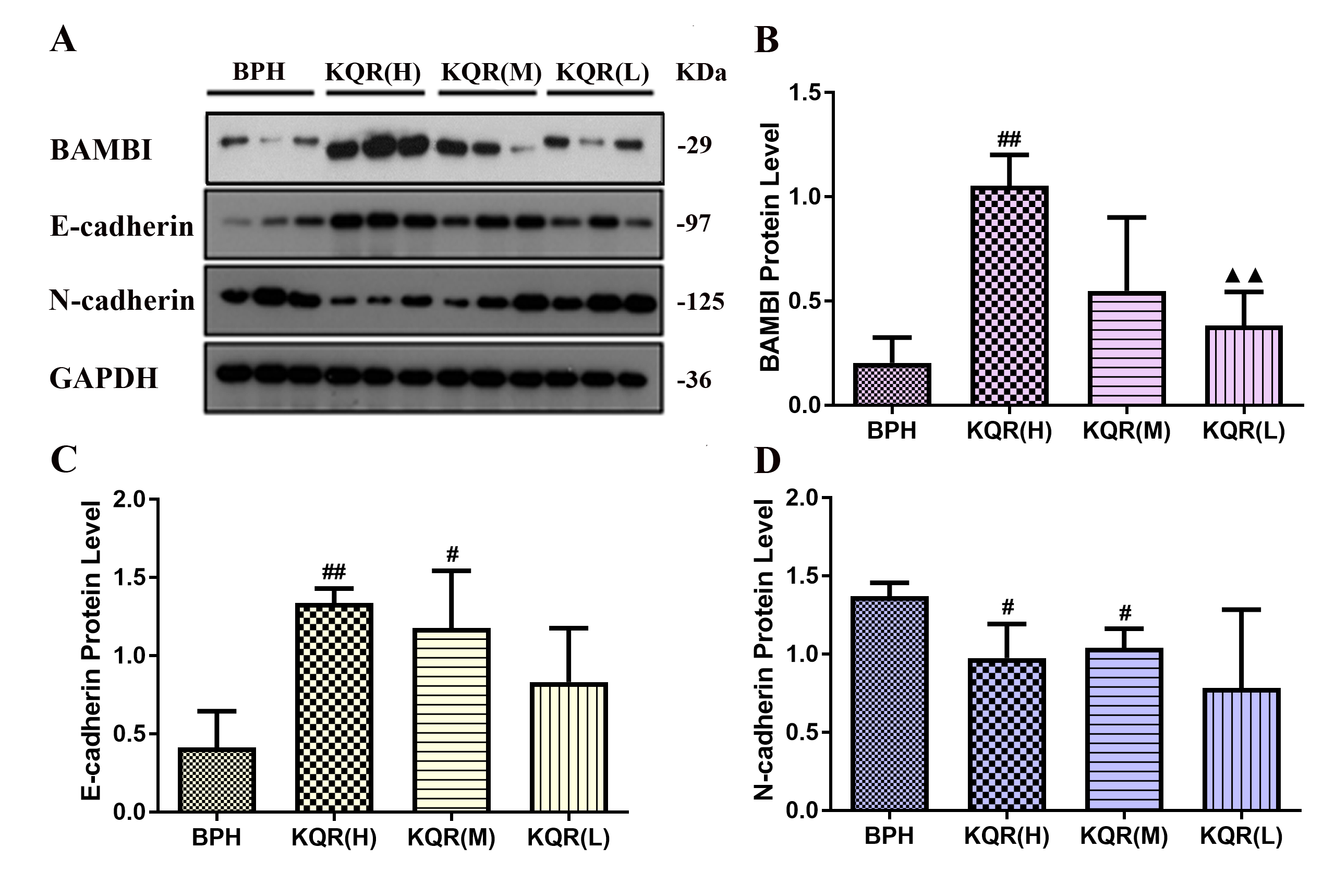


**Fig. S2.** Effects of KQR on the protein expression of BAMBI, E-cadherin and N-cadherin in the prostate tissue. Data were represented as mean ±SD (n=6 mice per group). Statistical analysis: ^#^*P*<0.05, ^##^*P*<0.01 compared with the BPH group; ^▲^*P*<0.05, ^▲▲^*P*<0.01 compared with the KQR high dose group.
